# Influence of Cr Ion Implantation on Physical Properties of CuO Thin Films

**DOI:** 10.3390/ijms23094541

**Published:** 2022-04-20

**Authors:** Katarzyna Ungeheuer, Konstanty Waldemar Marszalek, Marzena Mitura-Nowak, Marcin Perzanowski, Piotr Jelen, Marta Marszalek, Maciej Sitarz

**Affiliations:** 1 Faculty of Computer Science, Electronics and Telecommunications, AGH University of Science and Technology, 30-059 Krakow, Poland; marszale@agh.edu.pl; 2 The Henryk Niewodniczanski Institute of Nuclear Physics Polish Academy of Sciences, 31-342 Krakow, Poland; marzena.mitura-nowak@ifj.edu.pl (M.M.-N.); marcin.perzanowski@ifj.edu.pl (M.P.); marta.marszalek@ifj.edu.pl (M.M.); 3 Faculty of Material Science and Ceramics, AGH University of Science and Technology, 30-059 Krakow, Poland; pjelen@agh.edu.pl (P.J.); msitarz@agh.edu.pl (M.S.)

**Keywords:** ion implantation, cupric oxide, thin film, solar cell absorber

## Abstract

Cupric oxide is a semiconductor with applications in sensors, solar cells, and solar thermal absorbers. To improve its properties, the oxide was doped with a metallic element. No studies were previously performed on Cr-doping using the ion implantation technique. The research goal of these studies is to investigate how Cr ion implantation impacts the properties of the oxide thin films. CuO thin films were deposited using magnetron sputtering, and then chromium ions with different energies and doses were implanted. Structural, optical, and vibrational properties of the samples were studied using X-ray diffraction, X-ray reflectivity, infra-red spectroscopy, Raman spectroscopy, and spectrophotometry. The surface morphology and topography were studied with ellipsometry, atomic force microscopy, and scanning electron microscopy. A simulation of the range of ions in the materials was performed. Ion implantation had an impact on the properties of thin films that could be used to tailor the optical properties of the cupric oxide and possibly also its electrical properties. A study considering the influence of ion implantation on electrical properties is proposed as further research on ion-implanted CuO thin films.

## 1. Introduction

**Figure 1:**
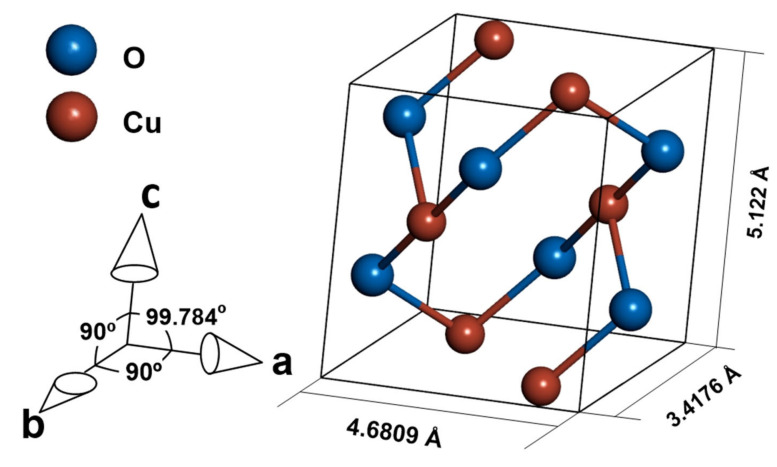
Crystal structure of CuO.

Cupric oxide is a p-type semiconductor with a wide range of applications, such as sensors [1,2,3,4], photocatalytic materials [5,6], diodes [7,8], and solar cells [9,10,11]. The material crystallizes in a tenorite structure of the monoclinic C2/c space group. (Figure 1) [12]. The semiconducting properties of CuO are due to Cu vacancies in the structure of the oxide [13]. The bandgap of cupric oxide is in the range of 1.2–1.9 eV [14,15,16], which is promising for solar cell application. According to Shockley–Queisser theory [17], the best possible efficiency is achievable for a material with a bandgap equal to 1.34 eV, and while commonly used silicon is limited by its 1.1 eV bandgap [18], CuO can have an optimal bandgap value [19], depending on production method and processing parameters [16]. Yet the types of band transitions in CuO strongly limit the possible efficiency of a solar cell made with a pure CuO absorber layer, as stated by Živkowić et al. [20]. To estimate the possible efficiency of a solar cell with CuO as an absorber, the SLME (spectroscopic limited maximum efficiency) metric [20,21] was used. Even assuming direct band gaps while indirect is the dominant type, the predicted efficiency does not exceed 3%. However, doping the material to change electrical properties or arrest recombination of carriers is a possible way to increase the efficiency of a CuO absorber [20].

CuO thin films can be modified to improve their properties or even achieve n-type conductivity. Yongli Du et al. obtained n-type CuO layers by the change of substrate temperature during a deposition in the process of magnetron sputtering [22]. S. Baturay et al. used the sol-gel technique and Co doping to obtain n-type CuO films and controlled the electric properties of the material by changing the amount of the dopant [23]. Solar cells with the structure of ITO/n-ZnO/n-CdS/p-CuO:Co/Au were made by L. Nkhaili et al. using a Co-modified CuO layer deposited with the RF sputtering technique. They obtained 0.46 V of open-circuit voltage, 4.1 mAcm^−2^ of short-circuit current, and a 30% fill factor [24].

One of the methods applied for semiconductor doping is ion implantation, which has been used for many years in the field of electronics [25]. It gives the possibility of introducing dopant at the desired depth of the material. U.D. Lanke and M. Vedawyas used Ar ions and improved the conductivity of CuO films [26]. The implantation was performed at 100 keV and with a dose of 5 × 10^12^ and 5 × 10^15^ ions/cm^2^. 

In this paper, we use ion implantation to introduce Cr ions into CuO thin films. The aim of this research is to determine the influence of ion implantation on the properties of cupric oxide thin films. Cr doping was proved to be beneficial for cupric oxide in sensor application. In their work, Szkudlarek et al. [2] prepared CuO:Cr thin films using DC-magnetron sputtering with a mosaic target to introduce Cr into the oxide. They also conducted calculations to study the electronic structure of the material and found that Cr doping decreases carrier concentration in the material and improves sensor response and selectivity towards acetone gas.

The cupric oxide was prepared by magnetron sputtering and next implanted in the following two series: at 15 keV and a dose of 5 × 10^16^ ion/cm^2^ and at 10 keV energy with doses of 1 × 10^14^, 5 × 10^14^, 1 × 10^15^ ion/cm^2^. The morphology of the films was studied using scanning electron microscopy (SEM), and optical properties were determined with spectroscopic ellipsometry and spectrophotometry. The thickness of deposited films was measured with X-Ray Reflectivity (XRR) and spectroscopic ellipsometry. The information about the structure of oxide before and after implantation was acquired with X-ray diffraction (XRD), infra-red, and Raman spectroscopy techniques. The predicted range of ions was calculated using the Stopping and Range of Ions in Matter (SRIM) program [27]. 

## 2. Results and Discussion

### 2.1. SRIM Simulations

**Figure 2:**
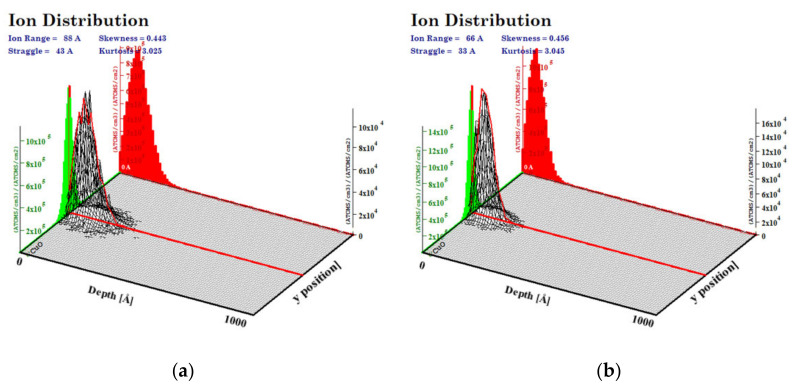
Range of implanted ions in CuO calculated with SRIM program for energy of (**a**) 15 keV, (**b**) 10 keV.

The simulations of the interactions of the ion beam with the CuO (a density of 6.00 g/cm^3^) were performed with the SRIM program for a 100 nm layer of oxide and a Cr ion beam of 10 keV and 15 keV energies. We calculated that the highest number of ions was implanted at a depth of 6.6 nm for 10 keV of Cr energy and 8.8 nm for 15 keV of Cr implantation energy. The maximum range of ions was higher, as follows: approximately 20 nm and 26 nm for lower and higher energy, respectively (Figure 2). In the simulations, the beam falls on the material surface perpendicular to its surface at a single point.

### 2.2. XRD and XRR Studies

**Figure 3:**
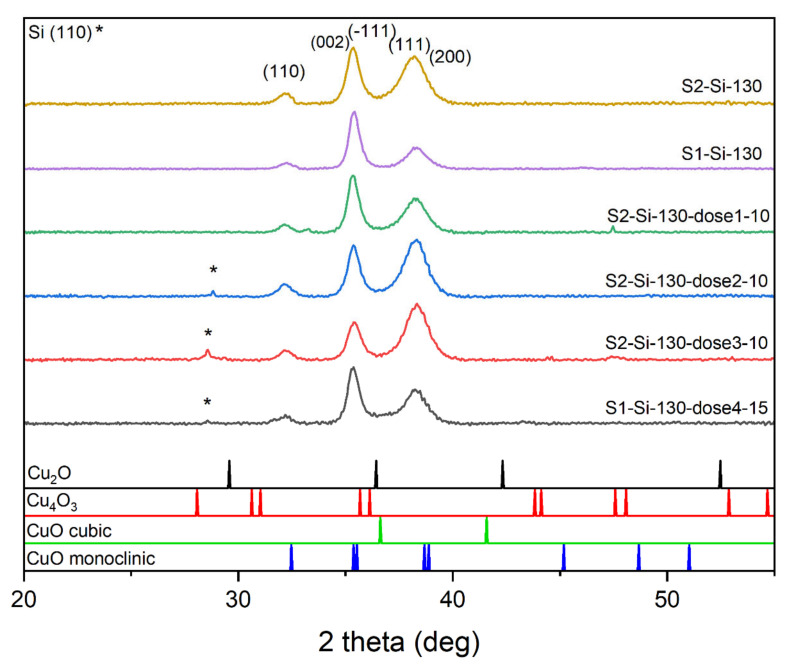
Diffractograms of the chosen CuO sample with the same thickness showing influence of different implantation dose.

We carried out XRD studies for all samples deposited on silicon. The diffractograms clearly show that the samples consist of a single CuO phase. For peak identification, PDF card no. 01-080-1268, 01-080-0076, 00-005-0661, and 00-041-0254 were used. Before the analysis, the background and the contribution from the characteristic radiation Kα2 of the Cu anode were removed from the XRD patterns using the X’Pert HighScore software. The most intensive peaks are (111) and (−111) combined with (002), while the (110) peak is also seen (Figure 3). The ratio of intensities of these two most profound peaks changes with implantation, which may suggest the presence of texturization of thin films. For the samples implanted with a lower energy of 10 keV and with a larger dose of ions, the intensity of the (002) + (−111) peak decreases while the intensity of (111) + (200) increases. When comparing peaks of S2-Si-130 and S2-Si-130-dose3-10, this intensity ratio change is substantial, while when comparing peaks of S1-Si-130 and S1-Si-130-dose4-15, this change is much less visible. Implantation with higher energy, even with a higher dose, has less impact on film texturization.

**Figure 4:**
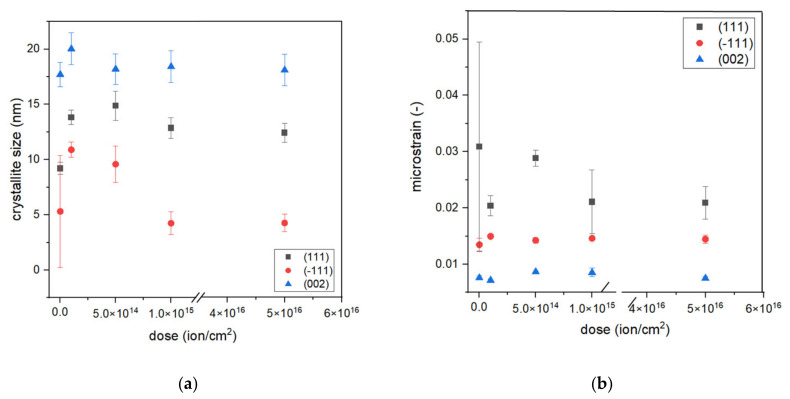
The dependence of (**a**) crystallite size and (**b**) microstrain on dose of implantation.

The (−111), (002), and (111) peaks were fitted using the pseudo-Voigt function, which is a weighted sum of Gauss and Lorentz functions as follows:

(1) $$y=y_{0}+A\left(m_{u}\frac{2}{\pi }\frac{w_{L}}{4{\left(x-x_{c}\right)}^{2}+w_{L}^{2}}+\left(1-m_{u}\right)\left(\frac{\sqrt{4ln2}}{\sqrt{\pi }w_{G}}e^{-\frac{4ln2}{w_{G}^{2}}{\left(x-x_{c}\right)}^{2}}\right)\right)$$

(2) $$\varepsilon =\frac{w_{G}}{4\tan\theta }$$

(3) $$D=\frac{\lambda }{w_{L}\cos\theta }$$

where *x* is an angle of Bragg peak position, *x*_c_ is an angle of the center position of the peak, *y*_0_ is an offset value, *A* is the area of the peak, *m*_u_ is the Lorentz fraction, *w*_L_ is the peak width of the Lorentz function, and *w*_G_ is the peak width of the Gauss function. Fitting allowed us to obtain *w*_L_ and *w*_G_ values that can be used to calculate crystallite size *D* and microstrain *ε* from Equations (2) and (3), respectively. The results of fitting and calculations of *D* and *ε* are presented in Figure 4. This peak fitting was performed for the following chosen samples: S1-Si-130, S1-Si-130-dose4-15, S2-Si-130-dose1-10, S2-Si-130-dose2-10, and S2-Si-130-dose3-10.

The crystallite size depends on their orientation. We observed that (−111) oriented crystallites (average size of around 7 nm) were smaller than (111) and (200) oriented crystallites, with an average size of 12 nm and 18 nm, respectively. The microstrain exhibits a different trend, the (−111) oriented crystallites demonstrate a larger strain than (002) crystallites but a smaller strain than (111). For all analyzed peaks, the crystallite size is the greatest for dose 2 of ion implantation, which is 5 × 10^14^ ions/cm^2^ at an energy of 10 keV. Comparing results responding to samples S1-Si-130-dose4-15 and S2-Si-dose3-10, it can be seen that both crystallite size and microstrain have almost the same values. This may suggest that the effect of a higher dose is diminished by the higher energy of ions.

The lattice constants a, b, c, and crystallographic angle of β of the CuO structure were calculated using the following formulas:

(4) $$\cos\beta =\frac{\sqrt{2}}{2}\left(\frac{1}{d_{-111}^{2}}-\frac{1}{d_{111}^{2}}\right)\frac{1}{\sqrt{\frac{1}{d_{-311}^{2}}-\frac{2}{d_{-111}^{2}}+\frac{1}{d_{111}^{2}}}}\frac{1}{\sqrt{\frac{1}{2d_{-111}^{2}}-\frac{1}{d_{110}^{2}}+\frac{1}{2d_{111}^{2}}}}$$

(5) $$a=\frac{2\sqrt{2}}{\sin\beta }\frac{1}{\sqrt{\frac{1}{d_{-311}^{2}}-\frac{2}{d_{-111}^{2}}+\frac{1}{d_{111}^{2}}}}$$

(6) $$c=\frac{1}{\sin\beta }\frac{1}{\sqrt{\frac{1}{2d_{-111}^{2}}-\frac{1}{d_{110}^{2}}+\frac{1}{2d_{111}^{2}}}}$$

(7) $$b=\frac{a\sin\beta d_{110}}{\sqrt{a^{2}\sin^{2}\beta -d_{110}^{2}}}$$

**Figure 5:**
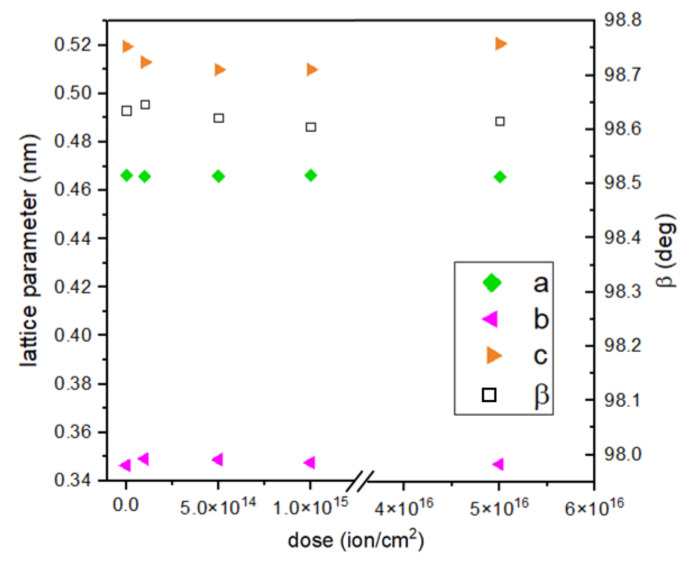
Calculated lattice parameters of CuO in dependence on dose of ions.

The calculated lattice constant *c* and crystallographic angle *β* do not seem to change substantially with the dose of implantation. The *c* constant value decreases with an implantation of lower energy and increases for the most intensive implantation. An opposite trend can be seen in the case of the *b* constant, which increases its value for doses 1, 2, and 3 and decreases for dose 4 (Figure 5). When comparing two samples with the highest ion doses and different energies, it is clearly noticeable that the *c* parameter has a higher value for 15 keV implantation. This parameter value for samples implanted with 10 keV of energy of Cr ions falls with increasing implantation, so energy seems to be the deciding implantation parameter here. 

To deposit a film of the desired thickness, first, a test deposition was performed with the same parameters as the final deposition. The thickness of the test deposition was measured with a profilometer to determine the time of the deposition needed to obtain a film of the desired thickness. For the second series of samples, the test deposition layer was 110 nm. Based on that, the deposition time was selected to obtain 130 nm layers of CuO. The X-ray reflectivity (XRR) measurements give information about the thickness of the samples, and the results differ from the assumed thickness (Table 1). This difference may be due to the specifics of the XRR method, which measures the signal from most of the sample area, while profilometry is a point measurement. Moreover, each deposition process may differ slightly from the test one. 

### 2.3. Surface Morphology and Topography

**Figure 6:**
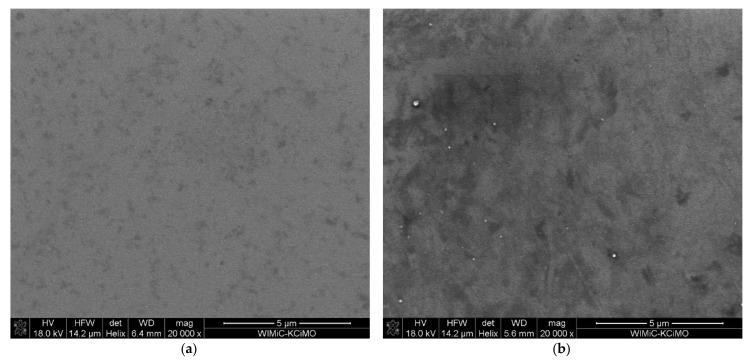
SEM images of sample (**a**) S1-Si-130 at 20,000× magnification, (**b**) S2-Si-130-dose3-10 at 20,000× magnification.

The SEM images of the ion-implanted samples shown in Figure 6 exhibit the granular structure of the films. The implanted sample (Figure 6b) has more dark areas visible, which indicates changes in the material and the possible presence of heavier elements, which in this case should be the implanted Cr.

The mean grain size was estimated using the ImageJ software [28], and we obtained values of 26 nm, 20 nm, 34 nm, and 21 nm for samples S1-Si-130, S2-Si-130-dose1-10, S2-Si-130-dose2-10, and S2-Si-130-dose3-10, respectively. The images used for grain size estimation were made with a 400,000× magnification. We demonstrated that ion implantation does not significantly change the film granularity. 

An energy-dispersive X-ray spectroscopy (EDS) measurement was made for series II samples. The measured EDS maps showed a uniform distribution of both elements, Cu and O, and no trace of Cr.

We measured with ellipsometric spectroscopy the maps of two non-implanted and implanted with the highest dose samples deposited on silica glass, and we determined the sample thickness and roughness. The central part of each sample was examined on an area of 5 mm × 5 mm with a 0.5 mm margin, resulting in an 81-point map. The data were fitted using the five Tauc–Lorentz oscillators model. The model was created by parametrization of the B-spline approximation. The average mean square error of the data fitting was 12.807 and 16.028, respectively, for non-implanted and implanted samples. 

**Figure 7:**
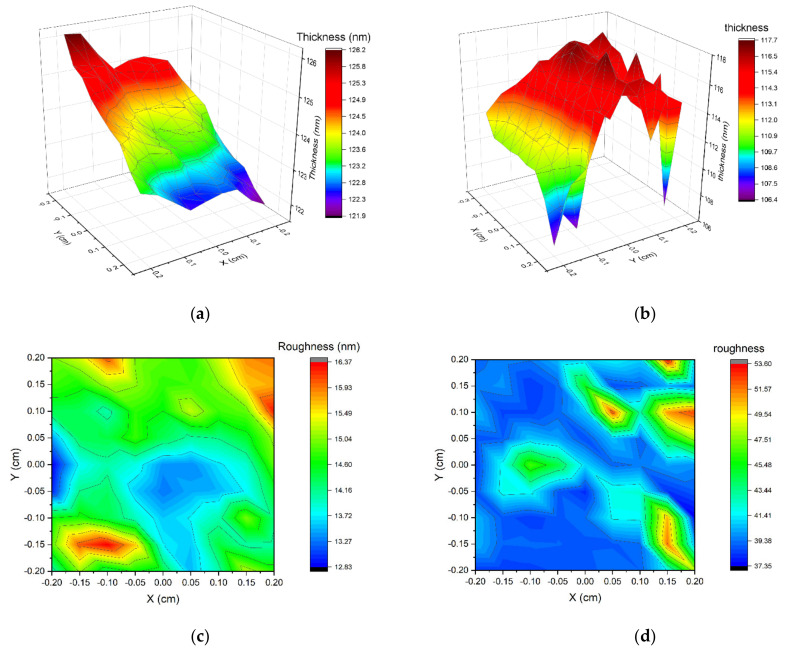
Thickness and roughness of two samples calculated from ellipsometric measurements. (**a**) S2-Si-130 (no implantation) thickness, (**b**) S2-Si-130-dose3-10 sample (dose of ions 1 × 10^15^ ions/cm^2^), (**c**) S2-Si-130 roughness, (**d**) S2-Si-130-dose3-10 roughness.

The measured area of the non-implanted sample shows a non-uniform thickness (Figure 7a, with a minimum value of 121.9 nm at one edge of the measured area and a maximum of 126.2 nm at the other edge. The average thickness is 123.8 nm, and the average roughness is 14.5 nm. The non-uniform thickness of this sample may be due to the substrate being placed still during the magnetron sputtering process. The implanted sample thickness shows different types of non-uniformity (Figure 7b), with no distinguishable slope. This sample has a minimum thickness of 106.4 nm and a maximum of 117.6 nm.

The roughness of implanted film is significantly higher than non-implanted, with an average value of 38.4 nm. This suggests that the ion implantation process increased the roughness of cupric oxide thin films. It was also observed by other researchers that ion implantation influences the roughness of material [29,30,31,32].

**Figure 8:**
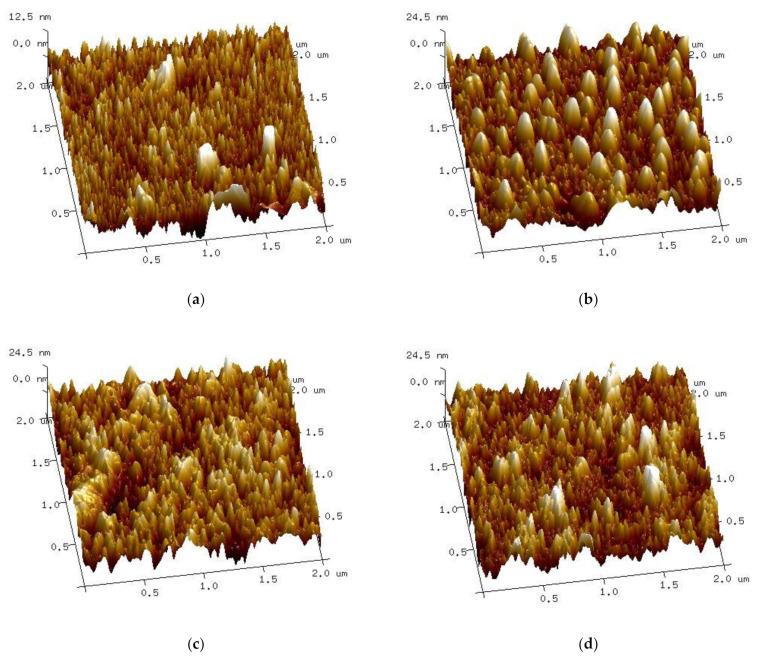
Atomic force microscopy 3D surface micrographs of samples (**a**) S2-Si-130, (**b**) S2-Si-130-dose1-10, (**c**) S2-Si-130-dose2-10, (**d**) S2-Si-130-dose3-10.

Atomic force microscopy gives the opportunity to study thin film surfaces with a higher resolution than the ellipsometric spectroscopy method. The roughness of the samples was estimated as the following two parameters: *R*_a_ (arithmetical mean deviation of the assessed profile) and *R*_max_ (maximum valley depth below the mean line). The roughness of non-implanted samples is the lowest at *R*_a_ of 2.36 nm and *R*_max_ of 39.7 nm. The values of implanted samples are significantly higher, as follows: *R*_a_ = 5.17 nm, *R*_max_ = 49.4 nm for dose 1, *R*_a_ = 5.79 nm, *R*_max_ = 54.9 nm for dose 2 and *R*_a_ = 5.16 nm, *R*_max_ = 57.1 nm for dose 3. The surface of the S2-Si-130-dose1-10 sample seems unusual compared to other samples (Figure 8b) as it shows regular curved objects. This sample also has a distinct result of XRD crystallite calculation, as it is characterized by bigger crystallites than the non-implanted sample.

Both ellipsometric and atomic force microscopy measurements prove that the ion implantation process increases the roughness of CuO thin films. These two methods give different results. It is important to notice that the AFM measurement covers a much smaller area (μm^2^) than the ellipsometric map (mm^2^). Moreover, the ellipsometric calculation is more prone to error as it requires the fitting of a model, while AFM is a direct measurement.

### 2.4. Optical Properties

**Figure 9:**
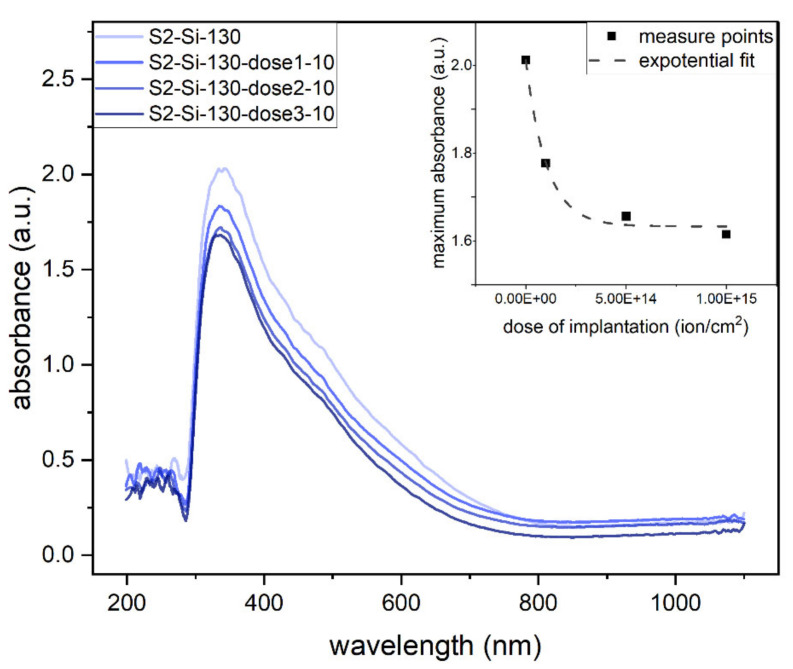
Absorbance of second series samples: CuO 130 nm on glass with different implantation at 10 keV. Inset: maximum absorbance as function of implantation dose.

The absorbance measurements clearly show that the absorbance decreases roughly exponentially with an increase in implantation dose, as shown in the inset of Figure 9. The maximum absorbance of the oxide thin films is in the UV range (Figure 9).

**Figure 10:**
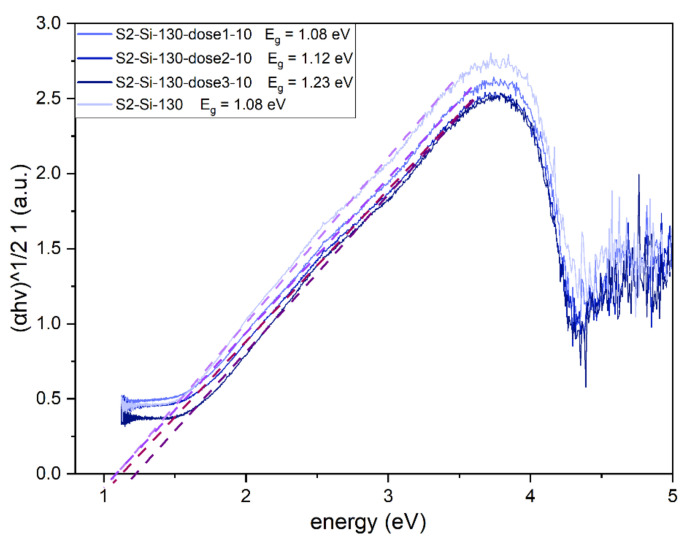
Tauc plot for II series samples with estimated indirect bandgap of CuO. The bandgap value increases with increasing dose of implantation.

The implantation has an impact on the optical bandgap of the CuO material, which has a direct and dominating indirect bandgap [15]. The Tauc plot was created for an indirect allowed transition (γ = 2). Implantation with Cr ions increases the bandgap from 1.08 eV for a non-implanted sample to 1.23 eV for a sample implanted with the highest dose (Figure 10).

### 2.5. Vibrational Properties

Vibrational measurements, Raman, and IR spectroscopy show that the spectra of samples after implantation exhibit fewer intensity bands than before implantation. This may indicate that the implantation process destroyed the CuO structure, either by lattice damage of the oxide or by amorphization of its structure. 

**Figure 11:**
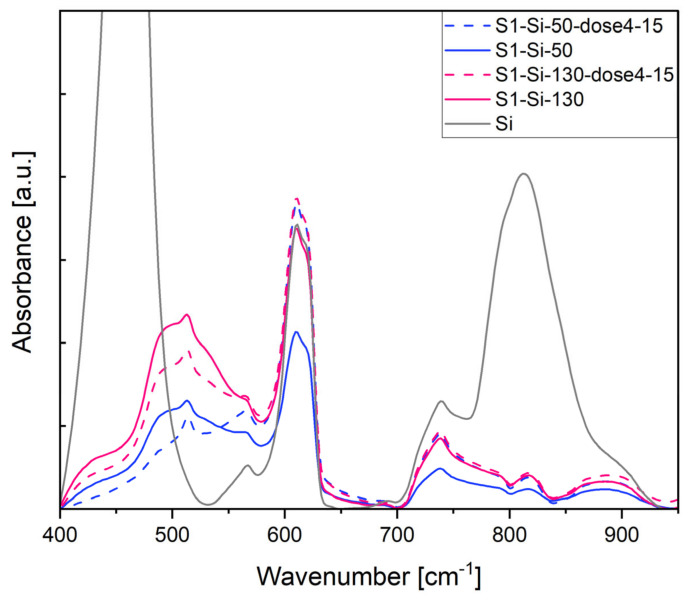
Absorbance of first series CuO samples on Si, measurement in IR transmission mode.

In Figure 11, the most pronounced band at 460 cm^−1^ comes from the Si substrate. A broadband around 500 cm^−1^ comes from the CuO structure and is a superposition of the following three IR active modes: A_u_ 457 cm^−1^, B_u_ 503 cm^−1^, B_u_ 568 cm^−1^, as determined by Debbichi et al. [33]. After implantation, the intensity of these bands decreases. 

**Figure 12:**
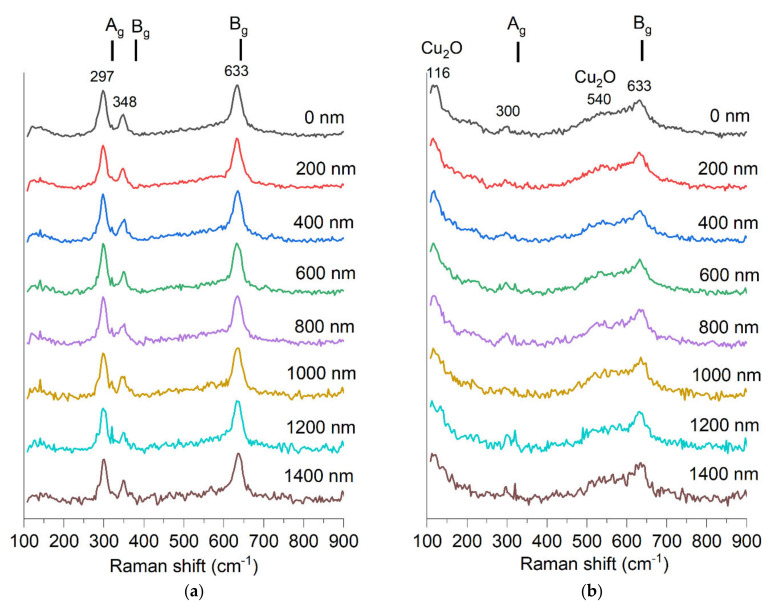
Raman spectra measured in depth of material with step of 200 nm of two samples (**a**) S1-G-1550 and (**b**) S1-G-1550-dose4-15.

The Raman spectrum clearly shows CuO bands for the non-implanted sample (Figure 12). The following three Raman active modes [33] are seen: A_g_ 319 cm^−1^, B_g_ 382 cm^−1^ B_g_ 629 cm^−1^. The sample after implantation shows only two bands with a significantly lower signal-to-noise ratio. It is known that ion implantation can induce a decrease in Raman band intensity [34]. The sample after implantation shows only two bands at 300 cm^−1^ and 633 cm^−1^, with a significantly lower signal-to-noise ratio. It is known that ion implantation can induce a decrease in Raman band intensity [34]. In the spectrum of the implanted sample, one more band is visible at 116 cm^−1^. CuO does not have any Raman active vibrational modes at this frequency shift. This band fits another copper oxide, Cu_2_O, which is an infra-red allowed mode, yet Raman spectroscopy results for Cu_2_O often show the presence of bands that theoretically should not be visible [35,36,37]. A broadband around 540 cm^−1^ also fits the Cu_2_O and corresponds to its only one Raman active vibration mode, T_2g_ [33]. This result shows that Cu_2_O may be present in this sample, though the XRD study showed only a peak for the CuO structure. The appearance of Raman bands corresponding to Cu_2_O may also indicate that the CuO oxide structure was altered by implantation and some non-stoichiometry emerged.

## 3. Materials and Methods

Thin films of CuO were deposited using magnetron sputtering on silicon, glass, and silica glass substrates. Before sputtering the substrates were cleaned with soap and water, rinsed with isopropanol and submerged in alcohol for 20 min of ultra-sonic bath, and finally dried with N_2_. Copper cathode (99.95% purity, Kurt J. Lesker, St. Leonards-on-Sea, East Sussex, UK) was used as target during sputtering. The power and current of discharge were 50 W and 80–130 mA, respectively. Before deposition of thin films, a presputtering process in Ar and then Ar+O_2_ was carried out. The pressure during deposition was kept at level of 1.5 × 10^−2^ mbar. The atmosphere in the chamber was 100% O_2_. Samples were made with different thicknesses of 30 nm, 55 nm, 130 nm, and 1550 nm. The thickness was measured with Taly-step profilometer of Rank Taylor Hobson for testing deposition processes. 

Ion implantation was performed at the Henryk Niewodniczanski Institute of Nuclear Physics Polish Academy of Sciences in Krakow. The source of ions was hydrated chromium trichloride. The implantation energy was 15 keV and the dose of ions was 5 × 10^16^ ion/cm^2^ for the first set of samples. The second set of samples was implanted with 10 keV energy and the following three different doses: 1 × 10^14^, 5 × 10^16^, and 1 × 10^15^ ion/cm^2^. The samples’ names and parameters are listed in Table 2. 

FTIR measurements were performed on the Bruker Vertex 70vspectrometer (Bruker, Billerica, MA, USA) in transmission mode, 256 scans with 4 cm^−1^ resolution, and in the range of 950 to 400 cm^−1^. Raman spectra were collected with WITec Alpha 300M+ spectrometer (WITec, Ulm, Germany) using 488 nm diode laser, 100× lens, with resolution of 3 cm^−1^ (lattice 600). Time of single measurement was 100 s–20 s per 5 accumulations.

Measurements of ellipsometric spectroscopy were performed at J.A. Woollam M 2000 ellipsometer (J.A. Woolam, Lincoln, NE, USA) at angles of 60°, 65°, 70°, and 75°. A map of ellipsometric measurements was prepared for the following two samples: non-implanted S2-Si-130 and implanted S2-Si-130-dose3-10. The maps were 5 × 5 mm^2^ with a step of 0.5 mm and 0.5 mm of margin. Spectrophotometry measurements of absorbance were performed with AvaLight-DH-S-BAL source and AvaSpec-ULS-RS-TEC (Avantes, Apeldoorn, The Netherlands) detector in transmission mode.

Structural properties of deposited oxide were studied with X-ray diffraction using PANalytical X’Pert PRO diffractometer (Malvern Panalytical, Malvern, UK) with Cu anode (0.154 nm radiation wavelength). The measurement step was 0.05° with time per step of 8000 s. On the same apparatus X-ray reflectivity measurements were performed to verify the thickness of the samples. The angular range was 0.2–3.0°, step was 0.003°, and time per step 10 s. 

Scanning electron microscopy observations were performed with NOVA NANO SEM 200 scanning electron microscope (FEI EUROPE COMPANY, Eindhoven, Netherlands) coupled with an energy dispersive spectroscopy (EDS) detector (EDAX).

The topography of tested samples was investigated using atomic force microscopy (AFM, Dimension ICON, Bruker, Santa Barbara, CA, USA) working in the tapping mode.

SRIM [28] calculation was made for silicon substrate with 130 nm layer of CuO. The parameters used in the simulation were the following: density of film 6 g/cm^3^, atomic ratio of Cu and O atoms 1:1, energy of Cr ions 10 keV and 15 keV, incident angle of ion beam 0°.

## 4. Conclusions

Thin films of cupric oxide were deposited on different substrates. The films were composed of only the CuO phase, as XRD, IR, and Raman spectroscopy measurements proved. Ion implantation with chromium ions had an impact on the structure and optical properties of the deposited oxide. Increasing doses of implantation resulted in deterioration of the crystal structure. The size of the lattice parameter changed with the higher energy of implantation, and the XRD peaks were less seen for implanted samples, suggesting changes in the crystal structure towards amorphization or crystallographic damage of the films. The ways dose and energy of implantation influence the structural properties of a material are different. No presence of Cr or its compounds was found with any of the used characterization methods. 

The optical properties of the oxide were also influenced by the ion implantation. The absorbance in the UV-Vis range decreased and the optical indirect bandgap value increased with a higher dose of ions. This shows the possibility to regulate the bandgap value by controlling the dose of implantation. The decrease in absorbance is not a promising effect for the absorber layer in thin-film solar devices, yet the utility of this approach needs to be reassessed considering the electrical properties of the films. 

Further steps can include measurements of the electrical properties of the films and a comparison of the oxide properties after annealing and diffusion of the Cr ions into the whole films. Moreover, a study to compare the effects of various energies of implantation may give interesting results. Thin films of CuO and modified CuO can find application in solar cells, sensors, or solar thermal modules.

## Figures and Tables

**Table 1:** Results of thickness measurements with XRR method.

Sample	Deposition Desired Thickness [nm]	XRR Thickness [nm]
S1-Si-55	55	68
S1-Si-55-dose4-15	55	-
S1-Si-130	130	152
S1-Si-130-dose4-15	130	96
S2-Si-130	130	119
S2-Si-130-dose1-10	130	127
S2-Si-130-dose2-10	130	-
S2-Si-130-dose3-10	130	114

**Table 2:** List of studied CuO samples with information about the substrate used during deposition and conditions of ion implantation.

Sample Name	Substrate	Energy of Implantation [keV]	Dose of Implantation [ions/cm^2^]
S1-Si-55	silicon	-	-
S1-Si-55-dose4-15	silicon	15	5 × 10^16^
S1-Si-130	silicon	-	-
S1-Si-130-dose4-15	silicon	15	5 × 10^16^
S1-G-1550	silica glass	-	-
S1-G-1550-dose4-15	silica glass	15	5 × 10^16^
S2-Si-130	silicon	-	-
S2-G-130	silica glass	-	-
S2-Si-130-dose1-10	silicon	10	1 × 10^14^
S2-G-130-dose1-10	silica glass	10	1 × 10^14^
S2-Si-130-dose2-10	silicon	10	5 × 10^14^
S2-G-130-dose2-10	silica glass	10	5 × 10^14^
S2-Si-130-dose3-10	silicon	10	1 × 10^15^
S2-G-130-dose3-10	silica glass	10	1 × 10^15^

## Data Availability

Data supporting this article are available upon request from corresponding author.
